# Eye and vision defects in under-five-year-old children in Oman: A public health intervention study

**DOI:** 10.4103/0974-620X.60015

**Published:** 2010

**Authors:** Rajiv Khandekar, Saleh Al Harby, Ali Jaffer Mohammed

**Affiliations:** Eye and Ear Health Care, Direcotrate of Health Services, Ministry of Health, Oman; 1 Director General of Health Affairs, Ministry of Health, Oman

**Keywords:** Childhood blindness, preschool screening, vision screening

## Abstract

**Purpose:**

To identify under-five-year-old children with vision or ocular defect in two provinces (Wilayats) of central Oman in 2006.

**Study Design:**

Public health intervention study.

**Materials and Methods:**

Ocular examination in Manah Wilayat was conducted by nursing staff of the primary health center (PHC) and in Mudhaiby Wilayat was conducted by a trainee Omani optometrist. Abnormal sized eyeball, strabismus, nystagmus and white pupil were recorded. Visual acuity was tested by LOGMAR chart with Lea's symbols in children >2 years of age and preferential viewing was assessed by Lea's grating paddle or 'Hiding Heidi' picture in children ≤2 years age. Data was analyzed using Statistical Package for Social Studies (SPSS 12).

**Result:**

Among 1,520 examined children, three children had absent eyeball bilaterally and three had unilaterally absent eyeball. Strabismus and nystagmus were detected in 44 (2.9%) and 18 (1.2%) children respectively. 'Hiding Heidi' test was normal in 530/537 (87%) of children. Distant vision reading was ≥0.32 in 386/448 (86.2%) eyes. Preferential looking test suggested that half of the children had defective vision (>2cpcm). Screening at '1-2 year' and '3-4 years' age group could significantly predict eye problems (P ≤ 0.001).

**Conclusion:**

Eye and vision screening of under-five kids helped in detection of eye problems in early stages. Instead of universal screening, high risk population or children of '3 to 4' years for vision and '1 to 2' years for ocular abnormalities is proposed The existing health services could not detect some children with eye problems and they were identified during such screening.

## Introduction

Childhood blindness is a priority eye problem in VISION 2020-The Right to Sight initiative.[1] Early detection of common and blinding eye ailments, especially in children is crucial for effective interventions. Vision screening is advocated when a child is enrolled in primary school (five to six years old).[2] However, low enrollment rates and lack of trained manpower in developing countries are barriers to such screening. Eye conditions like amblyopia, unilateral blindness and strabismus are often detected for the first time at this stage. Therefore, American Academy of Pediatrics recommends eye and vision screening for children under five years of age and suggests that early intervention will result in improved quality of life.[3] Before adopting this screening in developing countries, workload of such screening should be critically reviewed to ensure its efficiency and sustainability.

Oman has well-established primary health care (PHC) services, which are easily accessible to all. Any screening program targeting under-five children could be best implemented through PHC services. The child health care program, therefore, implemented developmental checks during a child's vaccination visits to the health institutes.[4] Eye examination to detect nystagmus, strabismus, cataract and absence of eyeball at specific ages is recommended by the eye health care program in Oman also.[5] Unfortunately, vaccination visits were found less suited for such developmental checks (Malankar P. Presentation 'Utilization of different sections of Child Health Card' in National Women and Child Care Program Workshop in 1998, Muscat, Oman).

Vision screening of children at school entry in Oman is conducted annually using Snellen's illiterate 'E' chart.[6] It is placed at six-meter distance from the child. The response of the child is essential to interpret visual status. By using the conventional methods, screening of younger children is difficult and often time consuming. Vision testing of younger children is now feasible using low cost portable tools that are used for low vision assessment.[7,8] Hence, we conducted a study to screen children under five to detect eye ailments and defective vision. The outcomes were expected to aid in establishing policies for eye screening of under-five children in Oman.

## Materials and Methods

This was a public health intervention study. The research and ethics committee of the Ministry of Health, Oman, provided written permission to conduct this study. We selected two health institutes with well-defined catchment areas namely Manah Health Center (HC) of Dhakhiliya region and Sinaw hospital of North Sharqiya region. The population in these areas was less likely to go to other health institutes for their basic health care. The under-five children residing in the above study areas were our study population. The study was conducted between September 2006 and December 2006. After announcement and campaigning in the study area, we enrolled children and invited them for eye examination at these two health institutes. If parents refused to participate, their children were excluded from the study.

In Manah HC, qualified nurses were trained in study methodology. They were involved in vision screening of school students. In Sinaw hospital, a trainee optometrist was our investigator. We used a pre-tested form and collected personal information like sex, date of birth, past history of visiting PHC services or ophthalmologist for eye problem and history of wearing spectacles. The field investigators examined eyes with 2.5 X ophthalmic loupe and torchlight. They were trained to detect rapid eye movement (nystagmus), white pupil and strabismus. The torchlight was held at half-meter distance from the face and the child was asked to look at the light. The reflection of light on cornea was observed. If it was not in the center of cornea, the child was considered to have strabismus.

Visual acuity testing was first attempted by using light box and distant vision chart with Lea′s symbol. If a child was already wearing spectacle, nurses tested him or her with the spectacles. The chart was kept at three-meter distance from the child. The children were explained the procedure with the help of optotype Lea's symbols. 'Pointing finger' method was used to test the visual acuity. LOGMAR values were used to record visual acuity of each eye. If child could not identify more than 50% of symbols correctly in a line, the visual acuity of that eye was considered to be of its upper line. If vision was better than 0.1, we considered that the vision was very good in that eye. If vision was under 0.3 LOG value in a 4-5 years old child and 0.4 LOG Value in a 3-4 years old child, the eye was considered to have defective vision. If child could not identify the symbols of the top line, the chart was brought nearer at one and half-meter distance and procedure was repeated.

If the child did not respond to the test using distant vision chart, investigators used Lea's grating paddle to determine the vision perception by the preferential looking method. In a child under two years, the visual acuity of 0.6 LOGMAR was considered normal. This test was conducted at one-meter distance. If the child moved eyes or face towards the paddle with strips, we considered preferential looking as 'present'. The paddle with smallest strips that the child could differentiate from the plain paddle was defined as his/her best visual acuity. We tested both eyes together in this screening. The notations were in cycles per Centimeter (cpCm). Vision was labeled normal if he/she could see stripes of two cpCm or more. If notations were under two cpCm, vision was considered to be defective. Infants not responding to the 'Lea paddles' due to young age, were shown 'Hiding Heidi' picture of 100% contrast and held at one-meter distance. If the child could see the picture of the doll and showed positive response, we considered preferential looking as 'present'.

Children with defective vision were rechecked after one week. If findings were different, the best visual acuity among two tests was noted. If parents had not consulted ophthalmologist in the past, they were referred to the ophthalmologists. If the child was already given eye care, parents were advised to consult their ophthalmologist periodically and approach the Ministry of Social Affairs for rehabilitation. We used 'EPI Data 2' software for data entry and Statistical Package for Social Studies (SPSS 12) software for data analysis. We calculated frequencies, percentage proportions and their 95% Confidence Intervals by using parametric method of univariate analysis.

The predictors of eye problems and defective vision in children were identified by binary logistic regression analysis. Dependent variables were 'children with eye problem' and 'children with defective vision'. Age group, gender, study area, history of consanguinity among parents, low birth weight (<1.5 kg), premature birth (<32 weeks) and birth order were the independent variables. We used 'step out method' in the regression model. Gender, study area and birth order were not statistically significant variables and hence were removed from the final regression table.

## Result

We examined 1,520 children (615 in Manah and 905 in Sinaw wilayat) in this study. Characteristics of examined children are given in Table 1. Proportion of children in different age group was not different. Parents of 522 (34.2%) children had consulted medical doctors at PHCs in the past for their child′s eye problem. Ophthalmologists had examined 240 (15.8%) children in the past and managed their eye problems.

**Table 1:** Characteristics of children screened for vision

	#	%
Gender		
Male	773	50.9
Female	747	49.1
Age group (year)		
<1	271	17.8
1 to 2	387	25.5
2 to 3	295	19.4
3 to 4	258	17
4 to 5	309	20.3
Study site		
Manah	615	40.5
Sinaw	905	59.5
Birth order		
1 to 3	1,228	80.8
4 to 5	108	7.1
6 to 10	129	8.5
11 to 15	26	1.7
Missing	29	1.9
Premature birth		
Yes	32	2.1
No	1,401	92.2
Missing	87	5.7
Low birth weight		
Yes	122	8
No	1,361	89.5
Dont't know	37	2.4
Total	1,520	

The ocular status of children is given in Table 2. We could identify 69 (4.5%) children with eye problems during the screening. The prevalence of bilateral blindness among children under five years of age was 2/1,000. Vision of each eye of children was noted on LOGMAR chart held at three-meter distance. But both eyes of a child were examined simultaneously when testing visual acuity with Lea's grating paddle and 'Hiding Heidi'. Children over two years responded to lea symbols on chart and matched with toy symbols. Under-two children accepted preferential looking tests better than vision chart testing methods. Vision was normal (>0.1) in (386/448) 86.2% of eyes of children examined with LOGMAR chart. We identified 323 (21.2%) children with defective vision using other methods [Table 3].

**Table 2:** Ocular status of under-five year old children in Oman

*Ocular status*	*# of children*	%
Absent eyeball		
Bilateral	3	0.2
Unilateral	3	0.2
Strabismus	44	2.76
Nystagmus	10	0.7
White pupil	12	0.8
Using spectacles on the day of screening	26	1.7

**Table 3:** Visual acuity of eyes by different methods in under-five children

*Distant vision LOGMAR*	*RE*		*LE*	
*(Snellen') (n = 204)*	#	%	#	%
–0.1 (6/4.8)	2	1	3	1.5
0 (6/6)	107	52.5	104	51
0.1 (6/9)	57	27.9	54	26.5
0.32 (6/12)	14	6.9	14	6.9
0.4 (6/15)	1	0.5	2	1
0.6 (6/24)	15	7.4	18	8.8
0.8 (6/38)	8	3.9	8	3.9
*Lea's grating paddle (cycle per cm)*	#	%
* * (n = 415;)		
8	153	36.9
4	129	31.1
2	40	9.6
1	40	9.6
0.5	45	10.8
0.25	26	6.3
Missing	18	4.3
Hiding heidi test for visual		
attention (n = 790)		
Defective	9	1.1
Normal	778	98.5
Missing	3	0.4

*Distant vision with LOGMAR chart with Lea's Symbol at 3-meter distance (Snellen's equivalent). If child was examined at 1.5 meter, the visual acuity was adjusted to 3 meter; **Test was conducted with both eyes open

At Manah Health Center, the health staff (doctor and nurse) identified 41 (6.1%) children with eye problems and suspected 149 (24.2%) children with defective vision. The optician of Sinaw hospital detected 28 (3.1%) children with eye problem and suspected 172 (19%) children with defective vision. The predictors of children with eye problems and defective vision along with adjusted Odd′s ratio, 95% Confidence Intervals and P values are given in Table 4.

**Table 4:** Predictors of ocular defect and defective vision in under-five children

*Variable*	Children with abnormal eye*			*Children with defective vision*		
	*Adjusted odd's ratio*	*95% confidence interval*	*P value*	*Adjusted odd's ratio*	*95% confidence interval*	*P value*
Birth before 32 weeks	1.75	0.37-8.34	0.48	1.03	0.43-2.50	0.81
Birth after 32 weeks	1			1		
Under 2 kg at birth	0.28	0.15-0.55	<0.0001	0.81	0.51-1.30	0.38
More than 2 kg at birth	1			1		
Age (years)
<1	2.23	0.84-5.96	0.11	0.21	0.12-0.34	1.3
1 to 2	2.85	1.17-6.96	0.02	0.27	0.18-0.41	7.1
2 to 3	1.89	0.71-5.02	0.2	0.96	0.68-1.37	0.83
3 to 4	3.99	1.62-9.83	<0.001	1.22	0.85-1.75	0.27
4 to 5	1			1		
Consanguinity in parents	0.25	0.14-0.46	5E-06	1.04	0.74-1.45	0.83
No consanguinity in parents	1			1		

Abnormal eye included nystagmus, absent eyeball, strabismus and white pupil

Low birth weight and history of consanguinity was negatively associated to the presence of abnormal eye (nystagmus, absent eyeball, strabismus and white pupil.) Birth weight, consanguinity among parents, age group '1 to 2 years' and '3 to 4 years' were significant predictors for the presence of eye problem in under five year old children. For defective vision we did not find a predictor of statistical significance.

## Discussion

Eye screening of children under five is conducted in many countries under varying names such as amblyopia screening program,[9-12] developmental checks for children[13] and strabismus detection in children of kindergarten.[3] However, the aim of such screening is common - to detect children with defective visual function and intervene at the earliest, to improve their quality of life.

Health services including free and easily accessible eye care are available to all citizens of Oman.[14] In spite of this, we observed that many children were detected with eye problems and defective visions for the first time in our study. This supported the recommendation that countries should adopt universal vision and eye screening for preschool children. Even the satisfaction of parents for timely detection of health ailments were less than desired.[15]

Two approaches were employed in this study using:

1. Existing health staff of a PHC and
2. A trainee optometrist.

We found that the latter had picked up more cases with eye problems compared to the PHC service staff. But in the absence of eye disease trends in two study areas, such comparison should be undertaken with caution. Family physicians are given the responsibility of primary eye screening of preschoolers.[16] PHC service staffs after brief training were also utilized for eye and vision screening.[17] Comparison of outcome of different staff used for vision screening suggested that they were uniformly less efficient compared to the tests in eye clinics with adequate tools.[18] Validity of vision screening and eye examination by public health nurse was found less satisfactory in <41 month old children but was good in three to four-year-old children.[19]

Our method of screening children of different age groups enabled us to determine trends of eye diseases and magnitude of defective vision in children under five. The screening method and the time taken for under three years old and three to five year-old children were different. Focusing on high-risk group or screening of children of particular age groups could improve efficiency of the screening. Predictors of eye problems that were identified in our study suggested that children of three to four years of age are high-risk group and should be screened for vision and anatomical defect. Similar observations were made in a study conducted in Canada. In our study, vision testing of children in young children was carried out with a method that was different from that applied for the three to five year-old children. In addition, field staff tested both eyes simultaneously of a young child with Lea's paddle and Hiding Heidi tests. A few cases of amblyopia could have been missed and therefore the prevalence of amblyopia could be an underestimate. Hence age 'one to two' predictor of visual defect, which we found in our study, should be looked with caution and confirmed by conducting a separate validity study of vision testing by preferential looking method with a larger sample.

As inter-observer validity among field staff was not conducted, a comparison of study results of two areas should be interpreted with caution. We suggest that a screening method with adequate validity be used as first level screening. Better training and monitoring could improve the screening results. However, the health staff should ensure that after first level screening the parents should be adequately counseled to improve the compliance for the follow up visits. Avoiding apprehension in parents about child's eye condition and stress on further consultations by experts should be the part of such counseling.

We did not have information on children suspected with eye problems and referred to ophthalmologists. This was due to non-availability of pediatric ophthalmologist in the study area. This limits us to present first level screening results only. For reliable estimates of strabismus, cataract and defective vision, further studies are recommended.

Absence of consanguinity among parents, normal birth weight in a child was associated with the presence of eye problems.[20] We could not explain the logic of such association. More studies with better methodology of generating information on these variables are recommended to confirm such association.

Such screening could be influenced by a learning curve of the staff, their commitment to perform such screening and cooperation of children and parents. To improve screening, Biagioli et al. suggest repeating the test after a few days and if results were consistently same, they proposed to take action.[21]

## Conclusion

Vision and eye screening conducted in a small sample of children of under-five years in Oman enabled us to detect children with eye problems for the first time in spite of having well established and accessible eye care services of primary and secondary levels within the reach of this community. Therefore, such screening is recommended. Instead of universal screening, high risk population or children of 'three to four' years for vision and 'one to two' years for eye problem is proposed to identify children with eye problem. Validity of eye screening of younger aged children should be established before recommending eye screening at larger scale.
